# An Integrated Pipeline for Combining *in vitro* Data and Mathematical Models Using a Bayesian Parameter Inference Approach to Characterize Spatio-temporal Chemokine Gradient Formation

**DOI:** 10.3389/fimmu.2019.01986

**Published:** 2019-10-11

**Authors:** Dimitris I. Kalogiros, Matthew J. Russell, Willy V. Bonneuil, Jennifer Frattolin, Daniel Watson, James E. Moore, Theodore Kypraios, Bindi S. Brook

**Affiliations:** ^1^Centre for Mathematical Medicine and Biology, School of Mathematical Sciences, University of Nottingham, Nottingham, United Kingdom; ^2^Department of Bioengineering, Imperial College London, London, United Kingdom

**Keywords:** chemokine transport dynamics, microfluidic device, model validation, Bayesian parameter inference, sequential Bayesian updating, MCMC methods, partial differential equations

## Abstract

All protective and pathogenic immune and inflammatory responses rely heavily on leukocyte migration and localization. Chemokines are secreted chemoattractants that orchestrate the positioning and migration of leukocytes through concentration gradients. The mechanisms underlying chemokine gradient establishment and control include physical as well as biological phenomena. Mathematical models offer the potential to both understand this complexity and suggest interventions to modulate immune function. Constructing models that have powerful predictive capability relies on experimental data to estimate model parameters accurately, but even with a reductionist approach most experiments include multiple cell types, competing interdependent processes and considerable uncertainty. Therefore, we propose the use of reduced modeling and experimental frameworks in complement, to minimize the number of parameters to be estimated. We present a Bayesian optimization framework that accounts for advection and diffusion of a chemokine surrogate and the chemokine CCL19, transport processes that are known to contribute to the establishment of spatio-temporal chemokine gradients. Three examples are provided that demonstrate the estimation of the governing parameters as well as the underlying uncertainty. This study demonstrates how a synergistic approach between experimental and computational modeling benefits from the Bayesian approach to provide a robust analysis of chemokine transport. It provides a building block for a larger research effort to gain holistic insight and generate novel and testable hypotheses in chemokine biology and leukocyte trafficking.

## Introduction

The precisely orchestrated migration of leukocytes plays a key role in all immune and inflammatory responses, including those that take place in infectious diseases. Their guidance to key destinations in tissues such as lymph nodes is coordinated by a group of small, secreted proteins called chemokines. Despite major recent advances in understanding chemokine functions (1–3), it is not yet clear how chemokine gradients are formed, maintained and regulated in tissues. A wide range of transport and biological processes contribute to the establishment, stabilization and regulation of chemokine gradients in interstitial tissue. These include e.g. chemokine production by endothelial cells in lymphatic vessels, chemokine diffusion and advection via interstitial fluid flow, chemokine binding to the extracellular matrix, scavenging of extracellular matrix-bound chemokine by atypical chemokine receptors expressed by macrophages or truncation of chemokines by dendritic cells. Dendritic cells exhibit both chemotaxis (by migrating up gradients of soluble chemokine) and haptotaxis (by migrating up immobilized chemokine gradients). Chemokine truncation or scavenging likely modifies the gradients as the leukocytes migrate, with the potential to affect subsequent leukocyte migration. Multiple cell types, competing interdependent processes and considerably uncertainty in both animal and *in vitro* models make for a system of such complexity that it cannot be understood using experiments alone (4–6). Mathematical models in combination with experiments can provide a way forward.

A full mathematical model represented by a system of partial differential equations [based on the original models of Keller and Segel (7)] accounting for all of the relevant processes results in a very large number of parameters, most of which have not been estimated from experiments. The predictive power of such mathematical and computational models relies critically on accurate estimates of these parameters. We have thus formulated a strategy to systematically estimate the parameters for the system. This requires the reduction of both mathematical model and corresponding experimental set-up to limit the number of parameters to be estimated at any one time. In this paper we have chosen to focus only on the transport processes associated with chemokine gradient formation. We present an integrated pipeline demonstrating the use of an advection-diffusion mathematical model in combination with measured spatio-temporal chemokine concentration profiles from microfluidic chambers in order to estimate the key transport parameters underlying the formation, development and establishment of chemokine gradients.

To provide a physiologically relevant environment for quantifying chemokine concentration profiles, we have designed a microfluidic chamber enabling the imaging and quantification of the diffusion of fluorescently tagged molecules from sources of low concentrations, similar to those measured *in vivo* for chemokines of 10–100 nM (8). Microfluidic chambers constructed of Polydimethylsiloxane (PDMS) provide a functional framework for both experimentally forming chemokine gradients and testing their effects on cultured cells. The devices can be imaged microscopically in real time. They feature a central hydrogel region lined by trapezoidal posts, which separate it from fluid channels into which chemokines are pumped. Previous designs have featured a space for deployment of extracellular matrix (ECM) bounded on either side by channels through which fluids containing cytokines can be pumped (9). Pressure differences across the hydrogel can be modulated to generate and control advection. The fluid velocity field across the hydrogel and diffusivity of chemokines within it need to be precisely known for model specification.

The purpose of this paper is to build a Bayesian framework that enables the estimation of these model parameters incorporating an assessment of the uncertainty in parameter estimation. In contrast to the classical frequentist inference approach, Bayesian methodology treats experimental data as a fixed quantity and parameters as random variables drawn from a probability distribution. This allows us to determine the probability of the parameters taking certain values given the observed data. Within this framework, we are able to incorporate prior knowledge about the probability distribution of the parameters which can then be updated through experimental observations. In addition, it allows for the assessment of the reliability of the parameter estimate through quantification of the uncertainty. This is a robust alternative to the traditional frequentist approach which deals with a single “best-fit” and confidence intervals based on potentially unrealistic assumptions in real experimental settings. Employing the Bayesian paradigm also facilitates the design of further experiments by demonstrating which experimental parameters have the greatest uncertainty. The suggested framework is validated by analyzing three datasets (hereafter referred to as DextranI and DextranII and CCL19), which capture the development of gradients of Dextran and CCL19 in microfluidic chambers.

## Materials and Methods

### Experimental Set-Up

**Figure 1:**
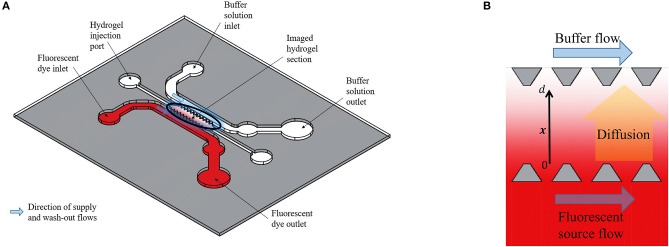
**(A)** Schematic representation of the polydimethylsiloxane (PDMS) microfluidic chip used for obtaining the experimental data. **(B)** Enlarged representation of the imaged hydrogel section between two open-ended channels. The Dextran diffuses from the one open-ended channel (source) to the other open-ended channel (buffer) and the fluorescent intensity across the distance *x*, with 0 ≤ *x* ≤ *d*, between the source and buffer (sink) fluid channels is recorded at fixed time steps. Based on the design of Farahat et al. (9).

The experimental data in this paper were obtained by microscopy imaging of Dextran and CCL19 transport in a polydimethylsiloxane (PDMS) microfluidic chip (Figure 1A). This chip enables the observation of the transport of fluorescently tagged solutes through a porous hydrogel (10). Here, the solutes were 10 kDa Dextran (ThermoFisher Sci., U.K.), which is of a similar molecular weight as the chemokines CCL19 and CCL21, and the chemokine CCL19 (Almac, U.K.). Both were labeled with the fluorophore Alexa^®^ 647 at one fluorophore per diffusing molecule and the hydrogel is collagen type I (Corning, U.S.A.) at 2.0 mg/mL. The fluorescent solution was supplied to an open-ended channel on one side of the hydrogel by means of a syringe mounted on a precision linear displacement mechanism (World Precision Instruments, model AL4002X). It was transported orthogonally to the supply flow direction into the hydrogel and was washed away by phosphate-buffered saline (PBS) on the opposite side of the hydrogel channel (Figure 1B). Dextran was supplied at a concentration of 100 nmoles/L, which is within the range of the concentration of bound CCL21 in lymph nodes *in vivo* and CCL19 was supplied at 25 nmoles/L, which is also within its concentration range in lymph nodes (8). The fluorescent intensity across the hydrogel was recorded at intervals of 30 or 120 *s* from an initial state of no fluorescence and averaged orthogonally using Fiji (11) with a custom Matlab code (MathWorks, Inc., U.S.A.). The fluorescence was also recorded across the source and sink fluid channels (Figure 1B) to provide boundary conditions for the posterior analysis.

### The Mathematical Advection-Diffusion Model

In this experimental set-up, the distance between the source and buffer (sink) of the microfluidic device (depicted in Figure 1B), is much larger than the gap between the trapezoidal structures at the side of each channel. Thus, we model the transport of Dextran and CCL19 in a one-dimensional domain 0 < *x* < *d* denoting the concentration of the solute by *C*(*x, t*) where *x* indicates the distance between the source and buffer with time denoted by *t* > 0. We assume that the supply of the solute at the source is approximately uniform along the channel, so that longitudinal variations are neglected. The transport of Dextran and CCL19 can, therefore, be described mathematically by the 1D unsteady advection-diffusion equation,

(1) $$\begin{array}{l} \frac{\partial C}{\partial t}=D\frac{\partial^{2}C}{\partial x^{2}}-u\frac{\partial C}{\partial x}\;,\;0<x<d, \end{array}$$

where *D* is the effective diffusivity (assumed uniform in the hydrogel) and *u* is the uniform advection velocity in the *x* direction, referred to as “advection” for the rest of the paper. Initial conditions for the concentration are extracted from the experimental data such that:

(2) $$\begin{array}{l} C\left(x,t_{0}\right)=C_{0}\left(x\right). \end{array}$$

We apply the following boundary conditions at the source and buffer:

(3) $$\begin{array}{l} C\left(0,t\right)=C_{s}\left(t\right)\text{ and }C\left(d,t\right)=C_{b}\left(t\right), \end{array}$$

with *C*_*s*_(*t*) and *C*_*b*_(*t*) specifying the measured time-varying concentration of solute (Dextran and CCL19) at the source and buffer, respectively. We solve Equations (1 − 3) numerically using a finite difference scheme. Central differences are used to discretize the diffusive terms of the equations and second-order upwinding is used for the advective terms. Time-stepping is performed using the implicit Euler method.

### Integration of Mathematical Model and Experimental Data in a Bayesian Framework

**Figure 2:**
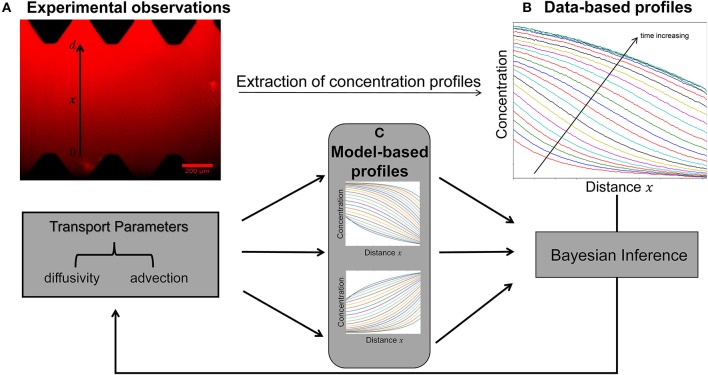
Schematic of the integrated pipeline for the estimation of transport parameters from the available experimental data. The data-based concentration profiles **(B)** at different time steps are extracted from raw images **(A)** using the image processing package Fiji. Sets of transport parameters of diffusion and advection enable the model simulations to generate concentration profiles at each time step **(C)**. The Bayesian inference approach is employed in order to determine this set of the candidate model parameters that best describes the experimental data by minimizing the discrepancy between the data-based **(B)** and model-based **(C)** concentration profiles at each time step.

**Figure 3:**
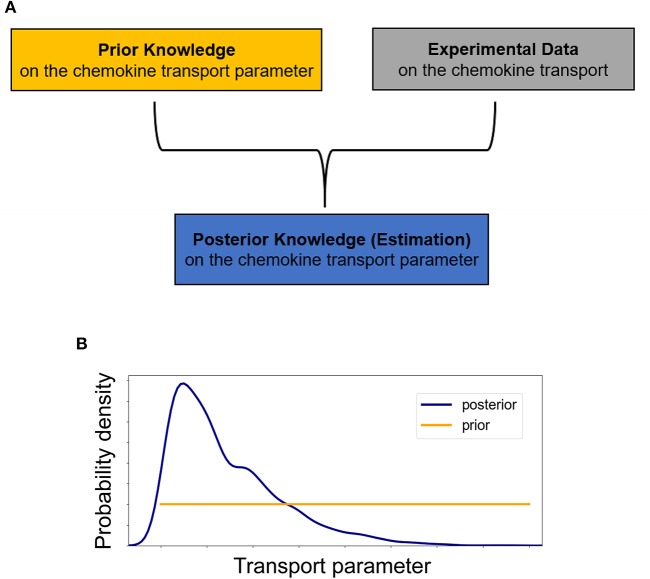
**(A)** Schematic of the essentials for employing a Bayesian approach in inferring transport parameters from experimental data. In the Bayesian paradigm, both transport parameters of diffusivity *D* and advection *u* are considered random variables and our prior knowledge of them is summarized into probability distributions, the *prior distributions*. The *experimental data* are used to update our prior beliefs about the transport parameters and lead to estimates of the transport parameters which include our data-informed knowledge in the *posterior distributions*. **(B)** Initially, we assume no prior knowledge about the transport parameter and thus we assign a vague (non-informative) prior distribution to it. Performing a Bayesian parameter analysis, we end up with a non-uniform posterior distribution which not only allows for a point estimate of the parameter but also provides a quantification of the uncertainty associated with it.

A key objective of this study is to quantify the parameters of diffusivity and advection from the available concentration profiles at each time step (Figures 2A,B). Estimation of model parameters consists of evaluating those values of the parameters which maximize the ability of the model (Figure 2C) to capture the experimentally observed concentration profiles (Figure 2B). We also aim to provide robust, quantitative information on the uncertainty associated with the estimated parameter values (Figure 3).

#### Experimentally Measured Initial and Boundary Conditions Incorporated in the Model

The crucial first step was to extract concentration profiles at each time point (Figure 2B) from time-lapse image data (Figure 2A) using Fiji (11). They were averaged over 300 μ*m* orthogonal to the main direction of diffusion and assimilated to fluorophore concentration using an assumption of proportionality between both values. The gray-scale profiles in the dataset at the first time step were used to determine the initial condition (Equation 2) for the mathematical model and the averaged gray-scale values closest to the source and buffer (sink) were used to generate the two boundary conditions (Equation 3) required for the mathematical model. However, the spatial grid and numerical time steps used to solve the discretized model equation do not necessarily coincide with the data points extracted from the imaging data. Therefore, it is convenient to find continuous approximations of the initial and boundary conditions from experimental data. We used linear interpolation for the initial conditions and fitted polynomials for the boundary conditions. Then, these are sampled at the relevant grid points and time steps used in the numerical method to provide the initial and boundary conditions for the model simulations. For each dataset, we evaluated polynomial fits for a range of orders and in each case we chose the lowest-order polynomial that gave a suitable qualitative fit to the experimental data.

**Figure 4:**
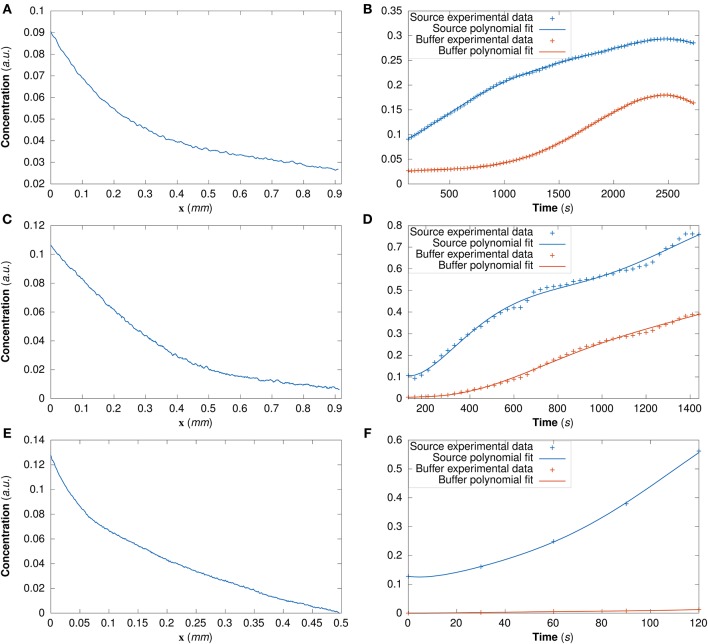
**(A)** The initial conditions for the model simulations extracted from DextranI **(A)**, DextranII **(C)** and CCL19 **(E)** through piecewise linear interpolation of the experimental concentration profile at each point along the channel of width 0.91 *mm* at the initial time *t*_0_ = 120 *s* for DextranI and DextranII and along the channel of width 0.496 *mm* at the initial time *t*_0_ = 0 *s* for CCL19. The concentration at the boundaries of the channel (the source and the buffer) was derived from DextranI **(B)**, DextranII **(D)** and CCL19 **(F)** (data points marked with crosses) by fitting polynomials of degree 5, 7 and 7 respectively (solid lines) to experimental data before being used as input to the model simulations.

For DextranI and DextranII, the initial conditions are derived from the experimental data at *t*_0_ = 120 *s* (Figures 4A,C); for CCL19 they are derived from the data at *t*_0_ = 0 *s* (Figure 4E). The time-varying boundary conditions are given by 5th order polynomials for DextranI (Figure 4B) and 7th order polynomials for DextranII and CCL19 (Figures 4D,F).

#### The Bayesian Paradigm

The main idea underlying the fitting of the model to data is to identify the parameters that best describe the observed concentration profiles (Figures 2B,C). If one were to use a traditional frequentist approach, the best estimates for the model parameters are those for which model and data outputs match as closely as possible, based on some objective function such as the sum of squared differences in the widely used “least squares” optimization technique. The frequentist approach asks the question—given a particular set of model parameters how well do the model solutions fit the experimental data? The Bayesian approach turns this question around: given the experimental data, what are the model parameters that best fit the observations? In addition, assessment of goodness-of-fit using frequentist approaches relies only on considering whether the data lie within some confidence intervals (with an underlying assumption that the model parameter estimates have an asymptotic Normal distribution). In contrast, the Bayesian approach enables the assignment of a probability distribution to the model parameters (which may or may not be a Normal distribution) and a quantification of the uncertainty associated with the fit (12).

We, therefore, adopt the Bayesian paradigm which enables us to (i) directly and satisfactorily assess the estimates of the model parameters given the observations already made in experiments and (ii) quantify the uncertainty of our estimates in a consistent, sound and intuitive probabilistic manner (13, 14). In order to fit the model described in Equation (1) to the fluorescence images at each time step, we assume additive Gaussian noise ε, independent for the experimental observations at each time step, with mean zero and standard deviation σ, i.e. ε ~ *N*(0, σ^2^), so that:

(4) $$\begin{array}{l} \bar{C}\left(x,t\right)=C\left(x,t\right)+\;\varepsilon , \end{array}$$

where *C*(*x, t*) indicates the model-based concentration and $\bar{C}\left(x,t\right)$ denotes the experimental data-based concentration at position *x* and time *t*.

Thus, at each time step both transport parameters of diffusivity *D* and advection *u* are considered random variables and our prior beliefs about them are formulated into probability distributions, referred to as prior distributions (Figure 3A). Based on Bayes' theorem, the experimental data are used to improve upon our prior belief by multiplying the prior distribution for each of the transport parameters by the likelihood, which describes the probability of a specific parameter value describing the observed data (Figure 3B) (15). After normalizing, this leads to the posterior distribution π(θ|*data*), i.e.,

(5) $$\begin{array}{l} \pi \left(\theta |data\right)=\frac{\pi \left(data|\theta \right)\pi \left(\theta \right)}{\int_{\theta }\;\pi \left(data|\theta \right)\pi \left(\theta \right)d\theta }\propto \pi \left(data|\theta \right)\pi \left(\theta \right),\text{for}\theta \in \left\{D,\;u\right\},\; \end{array}$$

where π(θ) signifies the prior distribution and π(*data*|θ) indicates the likelihood for each of the model transport parameters, i.e. the diffusivity *D* and advection *u*. However, in this study the uncertainty inherent in the experimental data, primarily caused by random error and its associated sources, was not measured directly in the observations and therefore the standard deviation σ of the noise ε also had to be estimated. This leads to the updated version of Equation (5), i.e.

(6) $$\begin{array}{l} \pi \left(\theta |data\right)=\frac{\pi \left(data|\theta \right)\pi \left(\theta \right)}{\int_{\theta }\;\pi \left(data|\theta \right)\pi \left(\theta \right)d\theta }\propto \pi \left(data|\theta \right)\pi \left(\theta \right),\text{for}\theta \in \left\{D,\;u,\;\sigma \right\}. \end{array}$$

#### Sequential Bayesian Inference of the Model Parameters

In order to accommodate the additional information provided by concentration profiles at different time points, we employ a sequential Bayesian approach. At the first time step, we assume no prior knowledge for the transport parameters of diffusivity *D* (*mm*^2^/*s*) and advection *u* (*mm*/*s*), while for the fluorescence imaging experimental noise some prior knowledge can be assumed. Specifically, at the start we assign a non-informative uniform prior distribution to both non-negative parameters of diffusivity *D* and advection *u* (Figure 3B) with 0 and 1 as their lower and upper bounds respectively, and a folded Normal distribution with mean zero (Half-Normal) to the non-negative standard deviation σ (arbitrary units based on fluorescence intensity). Thus, for the first time step:

(7) $$\begin{array}{l} D\;\sim \;\pi_{1}\left(D\right)=\;U\left(0,1\right), \end{array}$$

(8) $$\begin{array}{l} u\;\sim \;\pi_{1}\left(u\right)=\;U\left(0,1\right) \end{array}$$

and

(9) $$\begin{array}{l} \sigma \sim \pi_{1}\left(\sigma \right),\text{with }\sigma =\left|\sigma^{\prime }\right|\text{ and }\sigma^{\prime }\sim \;N\left(0,1\right). \end{array}$$

By updating the prior distributions π_1_(θ) through the likelihood function, which incorporates the information from the experimental data $E_{1}=\left\{\bar{C}\left(x_{i},t=t_{1}\right):0\leq x_{i}\leq d\right\}\;$ at the discrete points *x*_*i*_ at *t* = *t*_1_, Equation (6) leads to the posterior distribution $\pi_{1}\left(\theta |E_{1}\right)$ which summarizes the information for each parameter θ ∈ {*D, u*, σ} at the first time step, i.e.

(10) $$\begin{array}{l} D\sim \;\pi_{1}\left(D|E_{1}\right), \end{array}$$

(11) $$\begin{array}{l} u\sim \;\pi_{1}\left(u|E_{1}\right), \end{array}$$

and

(12) $$\begin{array}{l} \sigma \sim \;\pi_{1}\left(\sigma |E_{1}\right). \end{array}$$

At every subsequent time step *n*, with *n* ≥ 2, our knowledge of the parameter of diffusivity D, which is a characteristic quantity of the solute, is mathematically formulated in the prior distribution π_*n*_(θ) at the current time step *n* but it is also included in the posterior distribution $\pi_{n-1}\left(\theta |E_{n-1}\right)$ at the previous time step *n* − 1. We also assign a uniform prior distribution to advection *u*, which denotes the advection velocity, as we did for the first time step. Therefore, with the available experimental data $E_{n-1}=\left\{\bar{C}\left(x_{i},t=t_{n-1}\right):0\leq x_{i}\leq d\right\}$ at *t* = *t*_*n*−1_ we start afresh and write:

(13) $$\begin{array}{l} D\sim \pi_{n}\left(D\right)=\;\pi_{n-1}\left(D|E_{n-1}\right) \end{array}$$

and

(14) $$\begin{array}{l} u\sim \pi_{n}\left(u\right)=\;U\left(0,1\right), \end{array}$$

so that Equation (6) yields the following posterior distributions:

(15) $$\begin{array}{l} D\sim \;\pi_{n}\left(D|E_{n}\right), \end{array}$$

and

(16) $$\begin{array}{l} u\sim \;\pi_{n}\left(u|E_{n}\right). \end{array}$$

While the above holds for the parameter analysis of DextranII and CCL19 throughout the experiment, in the analysis of DextranI for time step *n*, with 2 ≤ *n* ≤ 6, in order to overcome the issue of parameter identifiability, we assign the posterior distribution at time step *n* − 1 as the prior distribution at time step *n* for the parameter of advection, i.e., $u\sim \pi_{n}\left(u\right)=\;\pi_{n-1}\left(u|E_{n-1}\right)$. Then, for any subsequent time step *n*, with *n* ≥ 7, Equations (14) and (16) hold, as explained above.

Since the noise in the fluorescence images was not measured directly, the prior distribution π_*n*_(σ) at any subsequent time step *n* for the standard deviation σ is given by:

(17) $$\begin{array}{l} \sigma \sim \pi_{n}\left(\sigma \right),\text{with }\sigma =\left|\sigma^{\prime }\right|\text{ and }\sigma^{\prime }\sim \;N\left(0,1\right), \end{array}$$

which gives rise to the following posterior distribution:

(18) $$\begin{array}{l} \sigma \sim \;\pi_{n}\left(\sigma |E_{n}\right), \end{array}$$

where $E_{n}$ indicate the available experimental concentration data at time *t*_*n*_. At the first time step, as described above, the initial conditions are extracted from the data. For any subsequent time step *n* ≥ 2, the initial conditions are updated using the values of the model parameters estimated through the sequential Bayesian approach which leads to a model-based concentration profile *C*(*x, t* = *t*_*n*−1_), at time *t* = *t*_*n*−1_.

#### Markov Chain Monte Carlo for Deriving the Posterior Distributions of the Model Parameters

The normalizing constant appearing in the denominator in Equation (5) is a multidimensional integral that can be cumbersome to determine analytically. Instead, simulation-based methods can be used for deriving the posterior distributions for each of the model parameters efficiently. In this study, we use a Markov Chain Monte Carlo (MCMC) algorithm (16) to efficiently generate samples from the posterior distribution which is considered the target distribution in our problem (17). We implement the widely-used random walk Metropolis-Hastings Algorithm (18, 19). The algorithms were implemented in the Python package PyMC which is intended for probabilistic machine learning and Bayesian stochastic modeling employing advanced Markov Chain Monte Carlo and variational fitting algorithms (20) using a Dell R720 with 2 x Intel(R) Xeon(R) E5-2665, 8-core processors and 512 Gb RAM.

The Metropolis-Hastings algorithm draws samples from the posterior distribution for each of the model parameters. Thus, we are able to summarize the posterior distribution and calculate the relevant statistical quantities of interest for each of the inferred parameters. These statistics include the mean, the median, the standard deviation and the Highest Posterior Density (HPD) intervals, which are the credible intervals in our Bayesian analysis.

At each time step *n* our prior knowledge for each transport parameter was updated through the posterior distribution at the previous time step *n*-1, as explained previously. However, the probability density functions of the posterior distributions resulting from the MCMC sampling are approximated well by a gamma distribution Γ(α, β), with the shape parameter α and the rate parameter β evaluated as follows (21):

(19) $$\begin{array}{l} E\left(\theta \right)=\frac{\alpha }{\beta } \end{array}$$

and

(20) $$\begin{array}{l} Var\left(\theta \right)=\frac{\alpha }{\beta^{2}}\;, \end{array}$$

with the mean *E*(θ) and the variance *Var*(θ) already known from the Bayesian statistical analysis for each transport parameter θ, with θ ∈ {*D, u*}.

## Results

**Figure 5:**
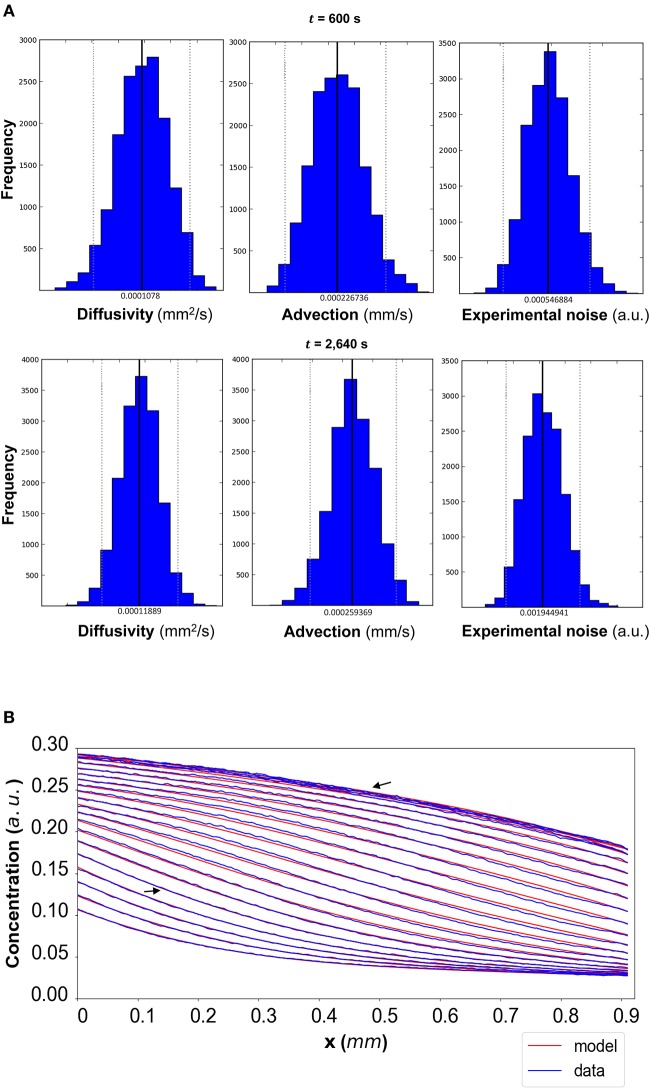
**(A)** DextranI: The posterior distributions for the diffusivity *D* (*mm*^2^/*s*), the advection *u* (*mm*/*s*) and the standard deviation σ (arbitrary units (a.u.) based on fluorescence intensity) shown for *t* = 600 *s* and *t* = 2,640 *s*. **(B)** The model-based concentration profiles *C*(*x, t*) with the median value of the resulting posterior distribution for each of the parameters as well as the data-based concentration profiles $\bar{C}\left(x,t\right)$ plotted every 120 *s* from 240 *s* to 2,640 *s*; the two concentration profiles annotated with an arrow correspond to those profiles resulting from the median values of the parameters whose posterior distributions are shown in **(A)**.

The results of the Bayesian parameter analysis provide us with posterior distributions for each model parameter at each time point. For DextranI, representative posterior distributions at *t* = 600 *s* and *t* = 2,640 *s* are shown in Figure 5A, for DextranII representative posteriors at *t* = 480 *s* and *t* = 1,440 *s* are depicted in Figure 8A and for CCL19 representative posteriors at *t* = 60 *s* and *t* = 120 *s* are given in Figure 11A. These plots show that the hereby presented analysis provides us not only with a single point estimate (the median values of the distributions) for each model parameter at each time but also enables us to quantify the uncertainty connected with each one of them. In fact, at a single time point these plots can interpret graphically all the summary statistics for each one of the inferred parameters *D*, *u*, σ contained in the Supplementary Material Tables 1–3 for DextranI, Supplementary Material Tables 4–6 for DextranII and Supplementary Material Tables 7–9 for CCL19. These summary statistics include measures of location (mean, median), measures of spread (standard deviation) as well as measures of confidence that the value of a parameter as estimated through its posterior distribution lies within a HPD (Highest Posterior Density) interval with 95% probability. Supplementary Material Tables 1–9 show that the values of median and mean for the model parameters consistently lie within the 95% HPD intervals at every time step. The Bayesian parameter analysis performed in this study satisfies certain convergence criteria (see Supplementary Material for results related to convergence, mixing and autocorrelation) thus allowing for efficient sampling of the posterior distribution for each model parameter at each time step.

In order to evaluate the predictability of the model and its ability to extract reliable values for the transport parameters, we use summary statistics of the posterior distributions of the estimated parameters as inputs into the mathematical model. Although following the analysis of the available datasets the median equals the mean of the posteriors for the vast majority of the time steps, we choose the median in order to account for the cases where the posterior distribution is skewed. The median values for each of the parameter distributions are then substituted in the mathematical model to simulate the concentration profiles (red curves in Figure 5B for DextranI, Figure 8B for DextranII, and Figure 11B for CCL19) corresponding to each time point for which *in vitro* concentration profiles were extracted (blue curves in Figure 5B for DextranI, Figure 8B for DextranII, and Figure 11B for CCL19). Figures 5B, 8B, 11B show that at each time step the inferred transport parameters lead to a very good overall fit of the model consistently for all datasets. While for DextranI and CCL19 the fit is excellent at all time steps, some discrepancies between the data-based and the model-based concentration profiles are more clearly detected in DextranII at *t* = 720 *s* and *t* = 1,200 *s* (Figure 8B). The difference at these time points is a result of the poor polynomial fit to the boundary conditions at the corresponding time points (Figure 4D).

**Figure 6:**
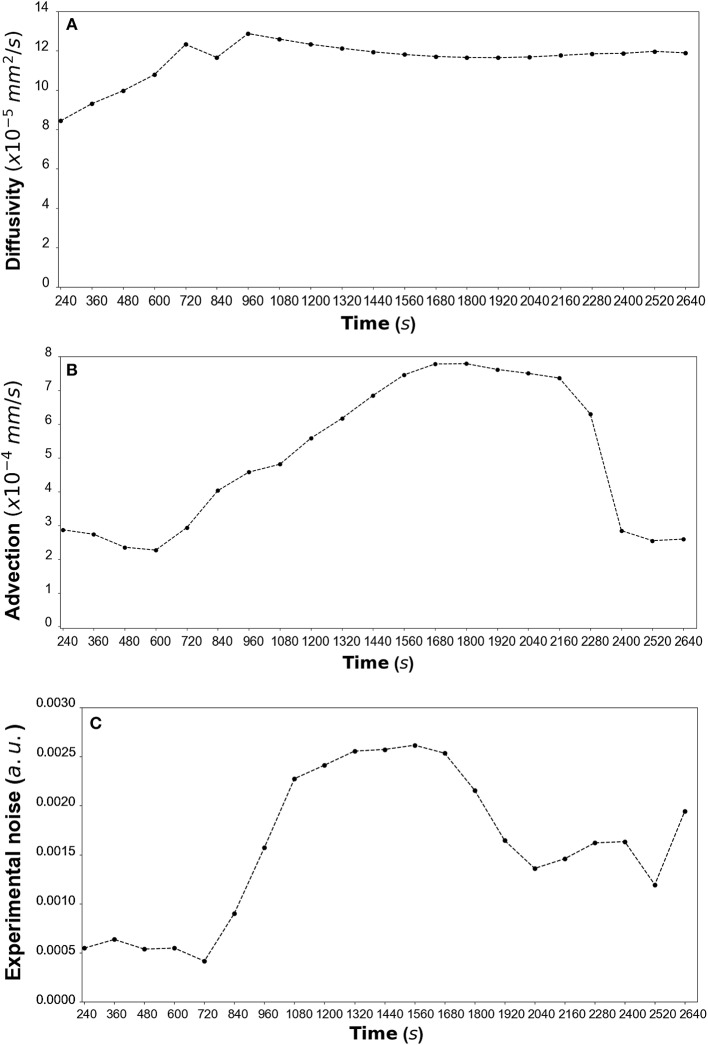
**(A)** DextranI: The estimated median values resulting from the posterior distribution for the diffusivity *D* (*mm*^2^/*s*) plotted against time every 120 *s* from 240 *s* to 2,640 *s*. **(B)** The estimated median values resulting from the posterior distribution for the advection *u* (*mm*/*s*) plotted against time every 120 *s* from 240 *s* to 2,640 *s*. **(C)** The estimated median values resulting from the posterior distribution for the standard deviation σ (arbitrary units (a.u.) based on fluorescence intensity) plotted against time every 120 *s* from 240 *s* to 2,640 *s*.

By fitting the model to experimental data at each time step we are also able to estimate the variation of the transport parameters over the course of the experiment (Figure 6 for DextranI, Figure 9 for DextranII and Figure 12 for CCL19). The median values of diffusivity varied between 10^−5^*mm*^2^/*s* and 10^−4^ *mm*^2^/*s* (Figures 6A, 9A, 12A). Based on the parameter estimation analysis, the advection across hydrogel varies over time (Figures 6B, 9B, 12B) due to limitations in the advection control in the microfluidic chamber.

**Figure 7:**
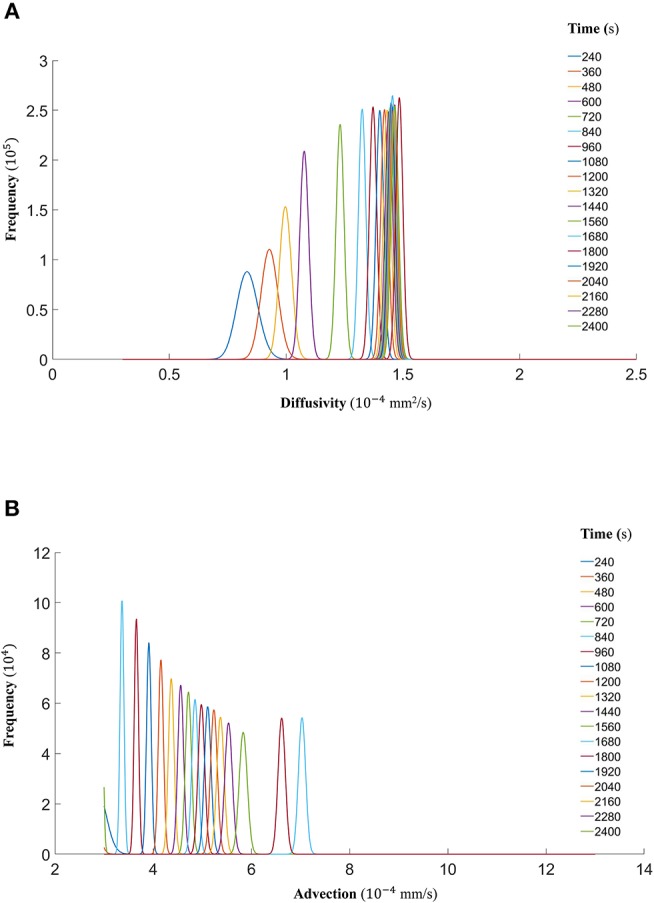
**(A)** The fitted gamma distributions to the posterior distributions of diffusivity *D* (*mm*^2^/*s*) at the different time points of DextranI. **(B)** The fitted gamma distributions to the posterior distributions of advection *u* (*mm*/*s*) at the different time points of DextranI.

Finally, we show that the probability density functions of the distributions are well approximated by a gamma distribution at each time step as explained in the Markov Chain section above. For all the datasets, Figure 7 (DextranI), Figure 10 (DextranII) and Figure 13 (CCL19) show the evolution of the posterior distributions for the estimated transport parameters of diffusivity and advection over the duration of the experiments. The range of the distribution at later time steps changes, because knowledge about the estimated parameter at the previous time step is incorporated by informing the prior distribution for the next time step. These figures also provide a sound argument to the above conclusion regarding the overall range of the diffusivity and advection over time guaranteeing that they are not distributed over multiple orders of magnitude.

**Figure 8:**
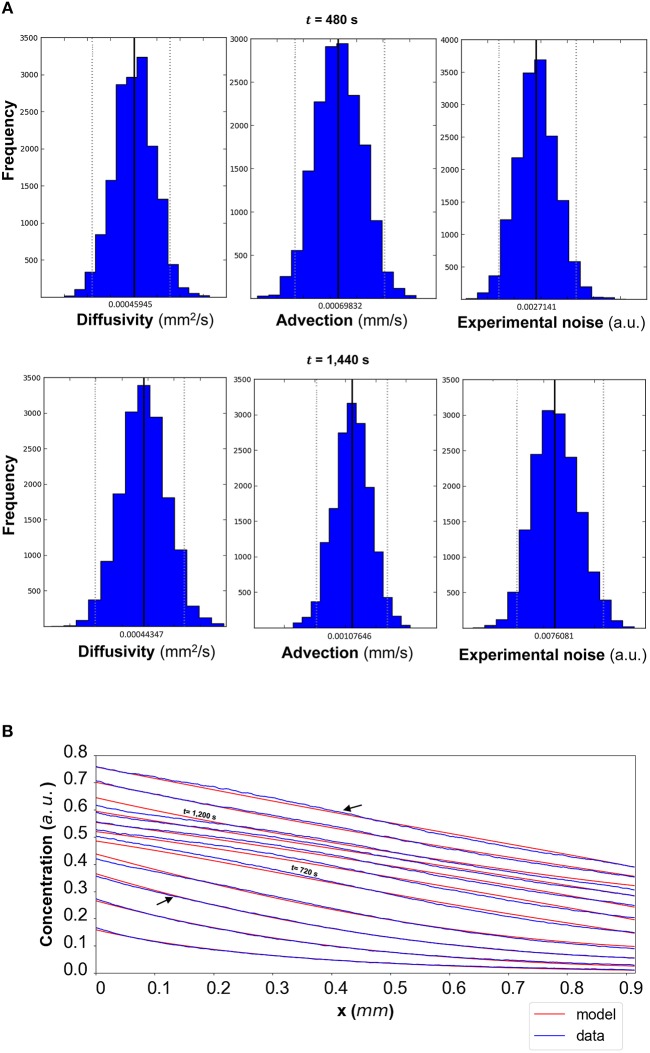
**(A)** DextranII: The posterior distributions for the diffusivity *D* (*mm*^2^/*s*), the advection *u* (*mm*/*s*) and the standard deviation σ (arbitrary units (a.u.) based on fluorescence intensity) shown for *t* = 480 *s* and *t* = 1,440 *s*. **(B)** The model-based concentration profiles *C*(*x, t*) with the median value of the resulting posterior distribution for each of the parameters as well as the data-based concentration profiles $\bar{C}\left(x,t\right)$ are plotted for each time step; the two concentration profiles annotated with an arrow correspond to those profiles resulting from the median values of the parameters whose posterior distributions are shown in **(A)**. The concentration profiles at *t* = 720 *s* and *t* = 1,200 *s* are also annotated.

**Figure 9:**
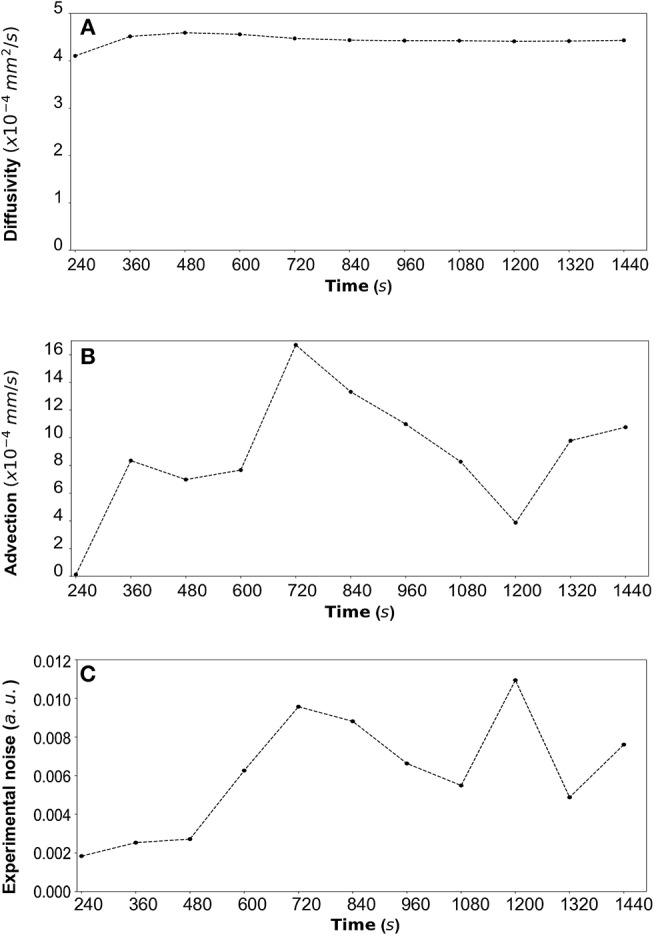
**(A)** DextranII: The estimated median values resulting from the posterior distribution for the diffusivity *D* (*mm*^2^/*s*) are plotted against time every 120 *s* from 240 *s* to 1,440 *s*. **(B)** The estimated median values resulting from the posterior distribution for the advection *u* (*mm*/*s*) are plotted against time every 120 *s* from 240 *s* to 1,440 *s*. **(C)** The estimated median values resulting from the posterior distribution for the standard deviation σ (arbitrary units (a.u.) based on fluorescence intensity) at each time step are plotted against time every 120 *s* from 240 *s* to 1,440 *s*.

**Figure 10:**
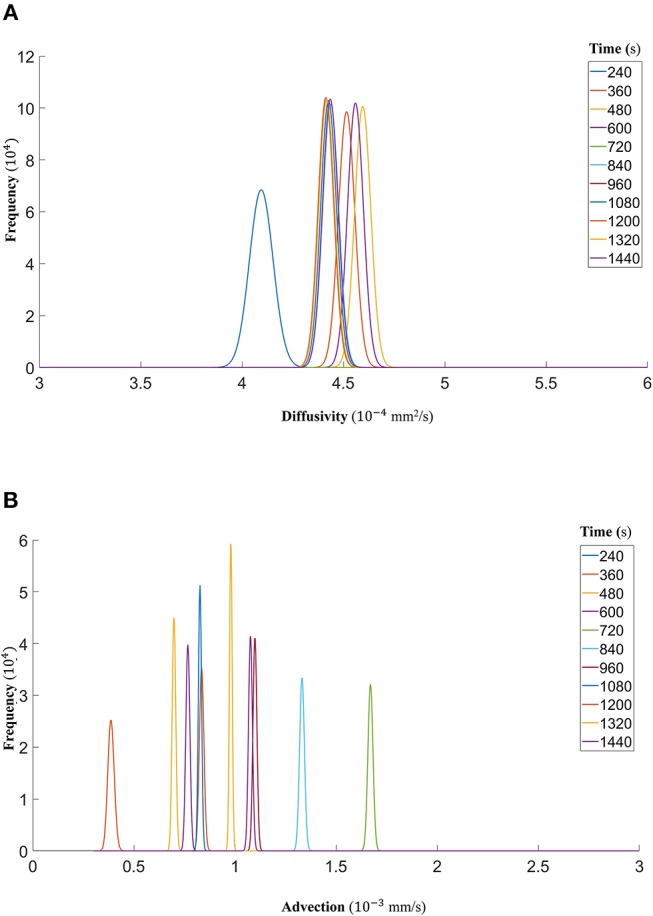
**(A)** The fitted gamma distributions to the posterior distributions of diffusivity *D* (*mm*^2^/*s*) at the different time points of DextranII. **(B)** The fitted gamma distributions to the posterior distributions of advection *u* (*mm*/*s*) at the different time points of DextranII.

**Figure 11:**
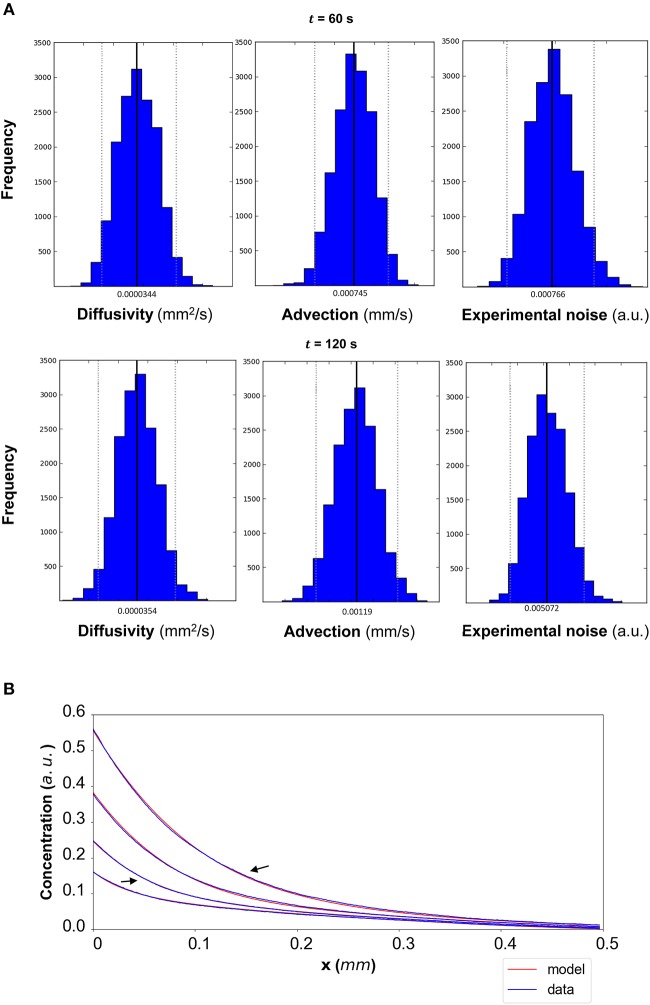
**(A)** CCL19: The posterior distributions for the diffusivity *D* (*mm*^2^/*s*), the advection *u* (*mm*/*s*) and the standard deviation σ (arbitrary units (a.u.) based on fluorescence intensity) shown for *t* = 60 *s* and *t* = 120 *s*. **(B)** The model-based concentration profiles *C*(*x, t*) with the median value of the resulting posterior distribution for each of the parameters as well as the data-based concentration profiles $\bar{C}\left(x,t\right)$ are plotted for each time step; the two concentration profiles annotated with an arrow correspond to those profiles resulting from the median values of the parameters whose posterior distributions are shown in **(A)**.

**Figure 12:**
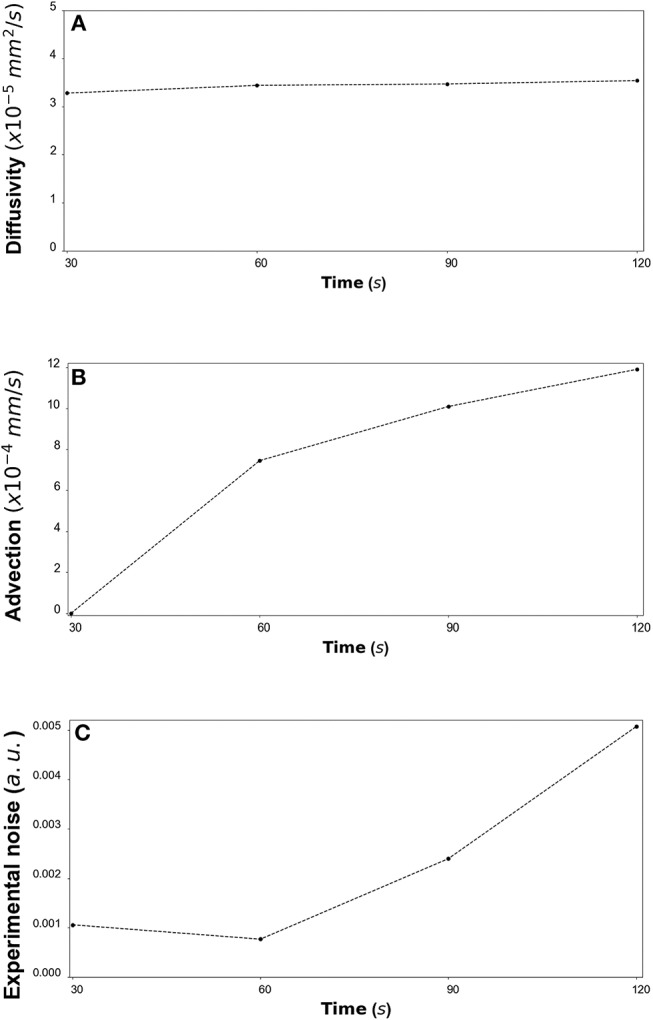
**(A)** CCL19: The estimated median values resulting from the posterior distribution for the diffusivity *D* (*mm*^2^/*s*) are plotted against time every 30 *s* from 30 *s* to 120 *s*. **(B)** The estimated median values resulting from the posterior distribution for the advection *u* (*mm*/*s*) are plotted against time every 30 *s* from 30 *s* to 120 *s*. **(C)** The estimated median values resulting from the posterior distribution for the standard deviation σ (arbitrary units (a.u.) based on fluorescence intensity) at each time step are plotted against time every 30 *s* from 30 *s* to 120 *s*.

**Figure 13:**
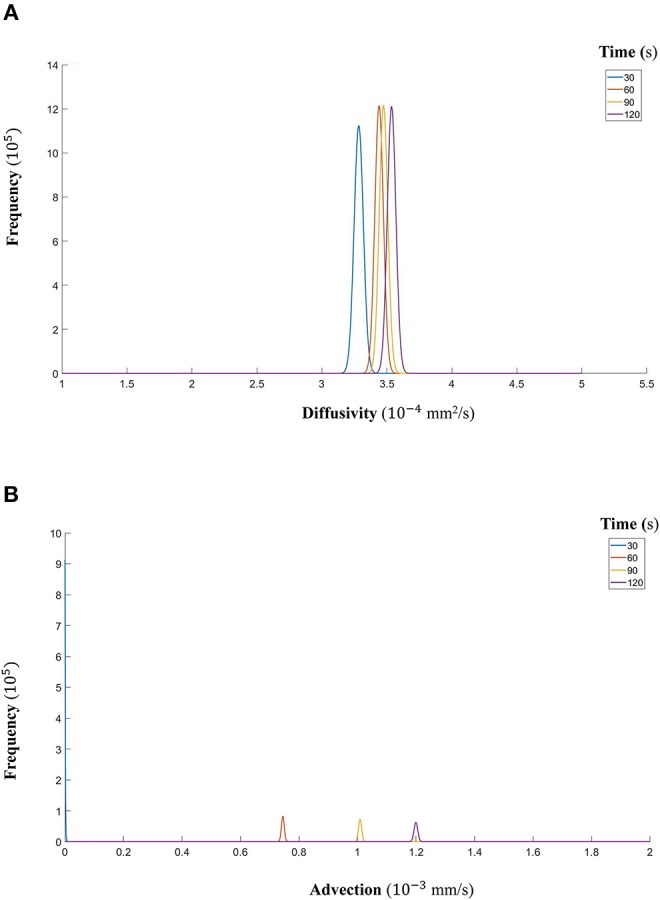
**(A)** The fitted gamma distributions to the posterior distributions of diffusivity *D* (*mm*^2^/*s*) at the different time points of CCL19. **(B)** The fitted gamma distributions to the posterior distributions of advection *u* (*mm*/*s*) at the different time points of CCL19.

## Discussion

This study illustrates a robust parameter estimation approach that greatly facilitates the use of mathematical modeling in extracting quantitative information about key mechanisms from experimental data in chemokine biology. The inclusion of biologically relevant parameters, including the statistically sound evaluation of their experimental uncertainty and variability, is crucial in modeling efforts to describe chemokine transport phenomena. This truly enables the model equations to represent the functional mechanisms in a manner that will appropriately represent the *in vivo* reality.

The example of parameter estimation shown here demonstrates an integrated pipeline for estimating key transport parameters from *in vitro* data using a mechanistic advection-diffusion model. The Bayesian framework not only produces an overall good fit of the model to the experimental datasets but it also allows for diffusivity and advection to be estimated robustly. The resulting estimations of diffusivity for Dextran varied between 10^−5^ and 10^−4^ *mm*^2^/*s* and were close to the values of diffusivity predicted or measured in other ways. Indeed, AL-Barati et al. (22) and Takanori et al. (23) measured the diffusivity to range from 10^−5^ and 10^−4^ *mm*^2^/*s* depending on the experimental conditions such as temperature. These values are also close to the Stokes diffusivity. Regarding the estimation of diffusivity of CCL19, these values are coherent with the theoretical Stokes diffusivity of 1.3 x 10^−4^ *mm*^2^/*s* for A647-labeled CCL19 in water, calculated for an average molecular weight of 11.5 kDa for the fluorescently labeled chemokines (manufacturer batch documentation). The effective diffusivity in porous media is expected to be up to an order of magnitude lower than this estimated value. Similarly, the order of magnitude of the advection velocity is 10^−4^ *mm*/*s*, i.e. a Péclet number lower than 1. This corresponds to the lower range of interstitial fluid velocities and is coherent with the fact that these data were obtained in devices intended for diffusive transport only. Because of the difficulty in balancing the system pressures, there was some variability in the advection velocity over time and this is captured by the parameter estimation algorithm. Diffusivity should not vary with time, so our estimates plateau out over time to the most representative value. The observation of advection variation over time is used in a feedback process for the refinement of the microfluidic chamber design. Its design aim is to enable precise and constant advection across the hydrogel, and the parameter estimations performed here help identify sources of error in the advection control strategy.

Fluorescence image noise is assumed to be independent for each time point, so it does not plateau. In addition, there were no data available about the fluorescence imaging experimental noise, which is quantifiable through the standard deviation σ (arbitrary units based on fluorescence intensity) as explained above and mathematically formulated in Equation (4). Although experimental noise is not known a priori (since we do not have multiple experimental repeats), our methodology enables us to estimate it. This is because our approach allows it to be treated as an extra parameter which can be inferred in tandem with both transport parameters successively throughout the duration of the experiment. The fact that our estimate for the noise was nominally about 1% of the fluorescence signal indicates that the data are of good quality.

This study also shows that Bayesian parameter analysis provides accurate posterior inference for all the estimated parameters at each time point during the course of the experiment. The framework provides point estimates of the three parameters of interest and assesses the uncertainty associated with each one by quantifying the corresponding statistical distribution. The resulting uncertainties in estimating diffusivity and advection are most likely a result of spatial variability due to hydrogel density variation and fluorescence imaging noise.

It is also worth noting that the initial and boundary conditions for the model simulations are extracted from the experimental data thus adding to the physical relevance of the estimated parameters of mathematical models and the reliability of the parameter inference approach itself. However, at certain time steps in one of the datasets (DextranII) the polynomial fit to the boundary condition fluorescence data was sufficiently poor to create disagreement with the model-based concentration profiles. Spline interpolation may be used as an alternative to address this issue.

The experimental set-up presented here is a prototype which only accounts for transport phenomena without incorporating binding kinetics. In future, the integrated pipeline for parameter estimation will be expanded to more complex experiments which also allow for binding kinetics, dynamic interactions between physical, biological, biochemical processes and cellular uptake. We will further perform experiments with different chemokines, as this could provide a broader understanding of chemokine gradient establishment and help stratify chemokines into relevant groups with respect to their gradient forming characteristics. This will also provide further support for the applicability and scalability of this integrated pipeline, since a quantitative understanding of a system with the complexity of chemokine transport dynamics requires not only a series of reductionist experimental approaches but also the ability to construct mathematical models with powerful prediction capabilities. The robust model parameter determination algorithm presented here provides the necessary foundation for this combined approach contributing to the emergence of a better knowledge base of the chemokine system and leukocyte trafficking. Thus, predictive modeling will provide invaluable insights into the potential therapeutic benefits of modulating immune response.

## Data Availability

Data will be made available on request.

## Author Contributions

TK, BB, and JM designed the study. DK and MR developed the code and performed the simulations. WB, JF, and DW conducted the experiments and extracted the data. All authors contributed toward manuscript writing and revisions.

### Conflict of Interest Statement

The authors declare that the research was conducted in the absence of any commercial or financial relationships that could be construed as a potential conflict of interest.
