# Supplementary material for: An Integrated Pipeline for Combining in vitro Data and Mathematical Models Using a Bayesian Parameter Inference Approach to Characterize Spatio-temporal Chemokine Gradient Formation
Source: Front Immunol. 2019 Oct 11;10:1986. doi: 10.3389/fimmu.2019.01986 (PMC6798077; doi:10.3389/fimmu.2019.01986)

Supplementary Material

Tables

Table 1 Summary statistics of the Bayesian analysis: The mean, the median, the standard deviation and the 95% Highest Posterior Density (HPD) intervals of the posterior distributions of the parameter of diffusivity D (mm^2/s) at the different time points of DextranI.

$\boldsymbol{D}(mm^2/s)$					
**Time** (s)	**Mean**	**Median**	**Standard Deviation**	**Lower 95% HPD**	**Upper 95% HPD**
240	0.0000834	0.0000835	0.00000454	0.0000745	0.0000915
360	0.0000931	0.0000931	0.00000362	0.0000862	0.0000996
480	0.0000997	0.0000996	0.00000260	0.0000947	0.0001048
600	0.0001078	0.0001078	0.00000191	0.0001041	0.0001115
720	0.0001231	0.0001232	0.00000169	0.0001196	0.0001262
840	0.000116593	0.000116564	0.00000168	0.000113249	0.000119841
960	0.000128717	0.000128701	0.00000158	0.000125524	0.000131729
1080	0.000125853	0.000125904	0.00000156	0.00012277	0.000128843
1200	0.000123266	0.000123238	0.00000152	0.00012032	0.000126227
1320	0.000121233	0.000121229	0.00000152	0.000118476	0.000124317
1440	0.000119472	0.000119398	0.00000147	0.000116614	0.000122355
1560	0.000118023	0.000118053	0.00000144	0.000115268	0.000120779
1680	0.000117085	0.000117068	0.00000144	0.000114294	0.000119891
1800	0.000116567	0.000116558	0.00000142	0.000113877	0.000119427
1920	0.000116465	0.000116436	0.00000142	0.000113841	0.000119364

2040	0.000116843	0.000116829	0.00000139	0.00011401	0.000119484
2160	0.000117555	0.000117601	0.0000014	0.000114707	0.000120203
2280	0.000118414	0.000118448	0.00000137	0.000115527	0.000120967
2400	0.000118648	0.000118643	0.00000139	0.000116022	0.000121413
2520	0.000119667	0.000119668	0.00000137	0.000117159	0.000122437
2640	0.000118888	0.000118888	0.00000135	0.00011623	0.000121535

Table 2 Summary statistics of the Bayesian analysis: The mean, the median, the standard deviation and the 95% Highest Posterior Density (HPD) intervals of the posterior distributions of the advection u (mm/s) at the different time points of DextranI.

$\boldsymbol{u}$ (mm/s)					
**Time** (s)	**Mean**	**Median**	**SD**	**Lower 95% HPD**	**Upper 95% HPD**
240	0.0002895	0.0002894	0.00001650	0.00025707	0.000318959
360	0.0002740	0.0002736	0.00001140	0.0002527	0.0002958
480	0.0002349	0.0002351	0.000007470	0.00022010	0.000249339
600	0.0002268	0.0002267	0.000004950	0.00021675	0.000235912
720	0.0002936	0.0002934	0.000003930	0.00028590	0.000301165
840	0.000402912	0.00040293	0.00000414	0.00039489	0.000411171
960	0.000457656	0.000457613	0.00000521	0.000447611	0.000467592
1080	0.000480873	0.000480919	0.00000705	0.00046811	0.000495374
1200	0.000558488	0.000558151	0.00000704	0.000544304	0.000572373

1320	0.000617144	0.000616844	0.00000731	0.000602642	0.00063101
1440	0.000684207	0.000684257	0.00000758	0.000669488	0.000699008
1560	0.000745437	0.000745393	0.00000795	0.000730116	0.000760983
1680	0.000777557	0.000777631	0.00000857	0.000761396	0.000794929
1800	0.000778438	0.000778486	0.00000767	0.000763202	0.000792437
1920	0.000761391	0.000761394	0.00000627	0.000749171	0.000773468
2040	0.000749802	0.000749794	0.0000056	0.000739611	0.000760789
2160	0.000735975	0.000735965	0.0000064	0.000723443	0.000747996
2280	0.00062965	0.000629602	0.00000748	0.000615965	0.0006452
2400	0.000284086	0.000284278	0.00000785	0.00026815	0.000298456
2520	0.00025469	0.000254655	0.00000548	0.000244411	0.000265969
2640	0.0002592	0.000259369	0.00000914	0.000240707	0.000276493

Table 3 Summary statistics of the Bayesian analysis: The mean, the median, the standard deviation and the 95% Highest Posterior Density (HPD) intervals of the posterior distributions of the standard deviation σ (arbitrary units (a.u.) based on fluorescence intensity) at the different time points of DextranI.

$\boldsymbol{\sigma}$ (a.u.)					
**Time** (s)	**Mean**	**Median**	**SD**	**Lower 95% HPD**	**Upper 95% HPD**
240	0.000544941	0.000543333	0.00002120	0.000505874	0.000587227
360	0.000636	0.0006346	0.00002480	0.0005854	0.000684
480	0.000536981	0.000536218	0.00002200	0.000493657	0.000579915

600	0.000547384	0.000546884	0.00002170	0.000503151	0.000587820
720	0.000415002	0.000414069	0.00001680	0.000382675	0.000448297
840	0.000902165	0.000901541	0.0000369	0.000836462	0.000976178
960	0.001574645	0.001573149	0.0000637	0.001449764	0.001700126
1080	0.002278521	0.002275535	0.0000912	0.002111289	0.002464112
1200	0.002418177	0.002413038	0.0000961	0.002231172	0.002602885
1320	0.002557702	0.002556374	0.0001	0.002382319	0.002778229
1440	0.002579034	0.002573419	0.000101817	0.002390903	0.002781911
1560	0.002618663	0.002616545	0.00010253	0.002422353	0.00282256
1680	0.002537909	0.002535366	0.000101779	0.002346806	0.002733853
1800	0.002159937	0.002157116	0.0000854	0.001993623	0.002320295
1920	0.001645691	0.001644033	0.0000644	0.00153258	0.001784329
2040	0.001359124	0.001358976	0.000055	0.001252055	0.00146436
2160	0.001458947	0.001457902	0.0000577	0.001342514	0.001567481
2280	0.001623797	0.001619942	0.0000639	0.001502004	0.001750245
2400	0.001636754	0.001632423	0.000065	0.001505808	0.001759405
2520	0.001194533	0.001192589	0.0000471	0.00110573	0.001288402
2640	0.001947216	0.001944941	0.0000769	0.001791183	0.002086324

Table 4 Summary statistics of the Bayesian analysis: The mean, the median, the standard deviation and the 95% Highest Posterior Density (HPD) intervals of the posterior distributions of the parameter of diffusivity D (mm^2/s) at the different time points of DextranII.

$\boldsymbol{D}(mm^2/s)$					
**Time** (s)	**Mean**	**Median**	**Standard Deviation**	**Lower 95% HPD**	**Upper 95% HPD**
240	0.0004095	0.0004105	0.0000058	0.0003969	0.0004189
360	0.000451619	0.000451654	0.00000404	0.000443872	0.000459335
480	0.00045956	0.000459448	0.00000396	0.000452026	0.00046735
600	0.000455964	0.00045608	0.00000391	0.000447304	0.000462886
720	0.000447548	0.000447441	0.00000395	0.000439309	0.000455007
840	0.00044378	0.000443774	0.00000393	0.000436309	0.000451504
960	0.000442698	0.000442723	0.00000388	0.000435139	0.00045051
1080	0.000442675	0.00044266	0.00000387	0.000435136	0.000450131
1200	0.000441418	0.000441331	0.00000383	0.000434066	0.000449165
1320	0.000441834	0.000441889	0.00000385	0.000434204	0.000449442
1440	0.000443538	0.000443473	0.00000386	0.000436277	0.000451313

Table 5 Summary statistics of the Bayesian analysis: The mean, the median, the standard deviation and the 95% Highest Posterior Density (HPD) intervals of the posterior distributions of the parameter of the advection u (mm/s) at the different time points of DextranII.

$\boldsymbol{u}$ (mm/s)					
**Time** (s)	**Mean**	**Median**	**SD**	**Lower 95% HPD**	**Upper 95% HPD**
240	0.0000165	0.0000123	0.0000148	0.0000000	0.0000459
360	0.000835192	0.000835168	0.0000113	0.000811207	0.000855683

480	0.000698351	0.000698323	0.00000882	0.000681586	0.000715715
600	0.000767079	0.000767091	0.00001	0.000748123	0.000786833
720	0.001669991	0.001669807	0.0000124	0.001645209	0.001693882
840	0.001331566	0.001331847	0.0000119	0.001309347	0.00135509
960	0.001098749	0.00109884	0.00000963	0.001079801	0.001117496
1080	0.000826984	0.000827024	0.00000778	0.000811841	0.00084234
1200	0.000387125	0.000386889	0.0000158	0.000357845	0.000419097
1320	0.000979551	0.000979449	0.00000664	0.000966776	0.000992385
1440	0.001076614	0.001076461	0.00000959	0.001058399	0.001095265

Table 6 Summary statistics of the Bayesian analysis: The mean, the median, the standard deviation and the 95% Highest Posterior Density (HPD) intervals of the posterior distributions of the standard deviation σ (arbitrary units (a.u.) based on fluorescence intensity) at the different time points of DextranII.

σ (a.u.)					
**Time** (s)	**Mean**	**Median**	**SD**	**Lower 95% HPD**	**Upper 95% HPD**
240	0.0018365	0.0018363	0.0000469	0.0017404	0.0019242
360	0.002531924	0.00253059	0.0000681	0.002392386	0.002662487
480	0.002714532	0.002714129	0.0000719	0.002569612	0.002856637
600	0.006270662	0.006263522	0.000173895	0.00595461	0.006637646
720	0.009566027	0.009568188	0.0002598	0.009052577	0.01005662
840	0.008816974	0.008814287	0.000237561	0.008338477	0.009279969

960	0.006630297	0.0066295	0.000181939	0.006272422	0.006990076
1080	0.005490246	0.005488968	0.00014392	0.005195598	0.005757418
1200	0.010945481	0.010943842	0.000294726	0.010361958	0.011510313
1320	0.004879747	0.004877373	0.000128235	0.004637463	0.005136889
1440	0.007610105	0.007608122	0.000203406	0.00723402	0.008010589

Table 7 Summary statistics of the Bayesian analysis: The mean, the median, the standard deviation and the 95% Highest Posterior Density (HPD) intervals of the posterior distributions of the parameter of diffusivity D (mm^2/s) at the different time points of CCL19.

$\boldsymbol{D}(mm^2/s)$					
**Time** (s)	**Mean**	**Median**	**Standard Deviation**	**Lower 95% HPD**	**Upper 95% HPD**
30	0.0000328	0.0000328	0.000000355	0.0000322	0.0000335
60	0.0000344	0.0000344	0.000000328	0.0000338	0.0000351
90	0.0000347	0.0000347	0.000000327	0.0000340	0.0000354
120	0.0000354	0.0000354	0.000000329	0.0000347	0.0000361

Table 8 Summary statistics of the Bayesian analysis: The mean, the median, the standard deviation and the 95% Highest Posterior Density (HPD) intervals of the posterior distributions of the advection u (mm/s) at the different time points of CCL19.

$\boldsymbol{u}(mm/s)$					
**Time** (s)	**Mean**	**Median**	**Standard Deviation**	**Lower 95% HPD**	**Upper 95% HPD**
30	0.00000102	0.000000715	0.00000101	0.000000000345	0.00000306
60	0.00007448	0.00007448	0.00000483	0.0007349	0.0007534
90	0.001009	0.001009	0.00000549	0.00098	0.00102
120	0.001199	0.00119	0.00000629	0.001188	0.001212

Table 9 Summary statistics of the Bayesian analysis: The mean, the median, the standard deviation and the 95% Highest Posterior Density (HPD) intervals of the posterior distributions of the standard deviation σ (arbitrary units (a.u.) based on fluorescence intensity) at the different time points of CCL19.

$\boldsymbol{\sigma}(\mathrm{a.u.})$					
**Time** (s)	**Mean**	**Median**	**Standard Deviation**	**Lower 95% HPD**	**Upper 95% HPD**
30	0.00105843	0.001058312	0.0000268	0.001007237	0.00111213
60	0.0007665	0.0007662	0.0000199	0.0007252	0.0008029
90	0.00239702	0.00239702	0.0000635	0.002271	0.002517
120	0.005074	0.005072	0.000131	0.004796	0.005313

Traceplots

We monitor and assess the convergence of chains through the trace plots of samples against the simulation index over multiple runs of the Metropolis-Hastings sampling method for 35,000 total iterations with 19,000 burn-in iterations for the diffusivity D, the advection u and the standard deviation σ. The resulting chains are well-mixed and the obtained samples are effectively independent by monitoring the autocorrelation which measure the dependency among Markov chain samples. Some representative trace plots and autocorrelation plots are presented in the following pages.

Dataset I

Diffusivity $\boldsymbol{D}(mm^2/s)$

$t = 2040$ s

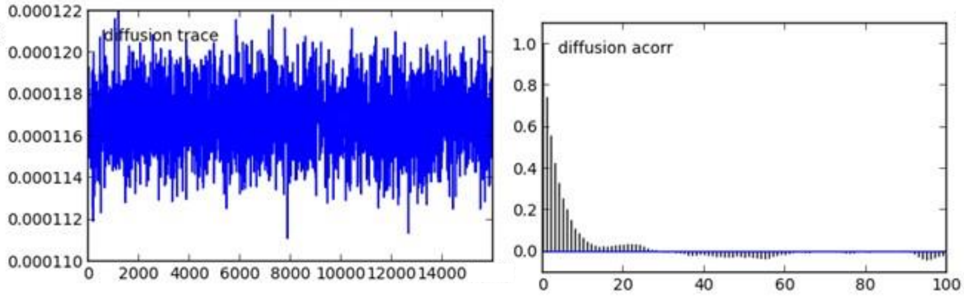

$t = 2160$ s

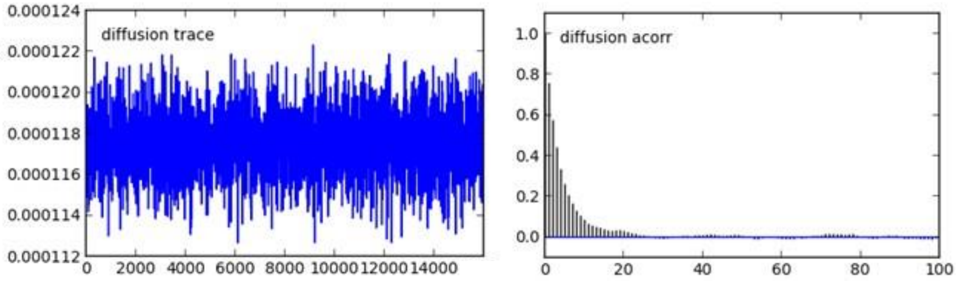

$t = 2280$ s

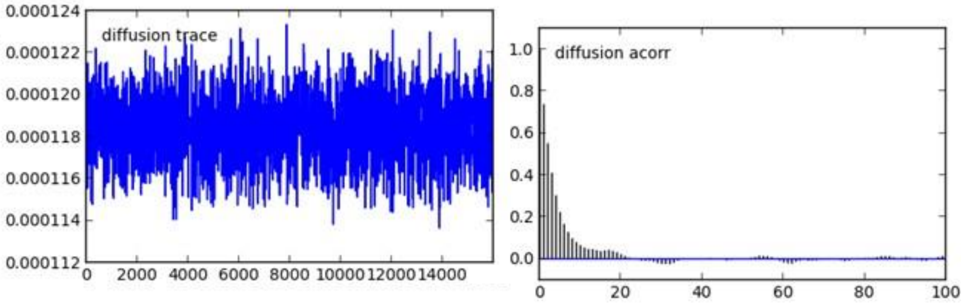

$t = 2400$ s

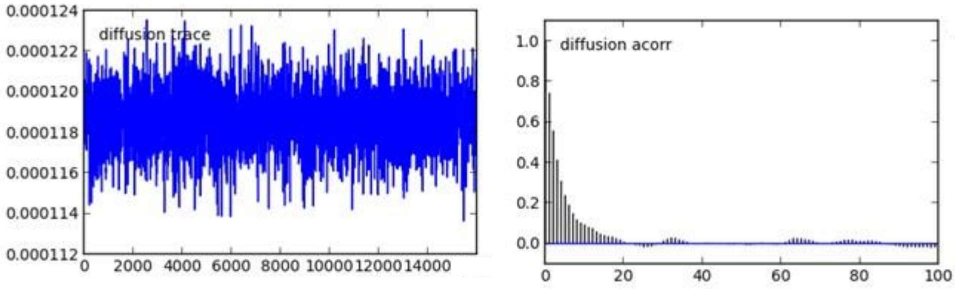

$t = 2520$ s

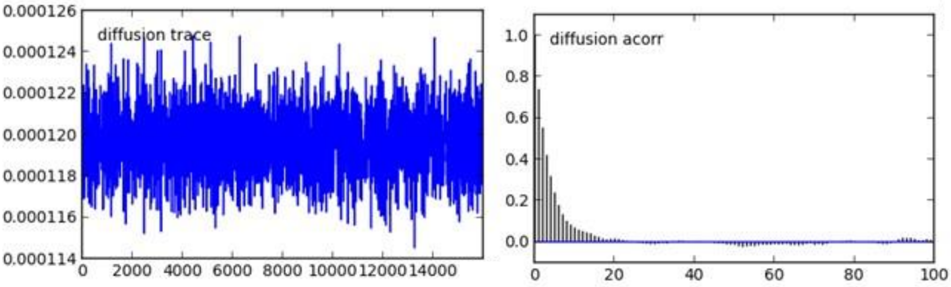

Dataset I

Advection u (mm/s)

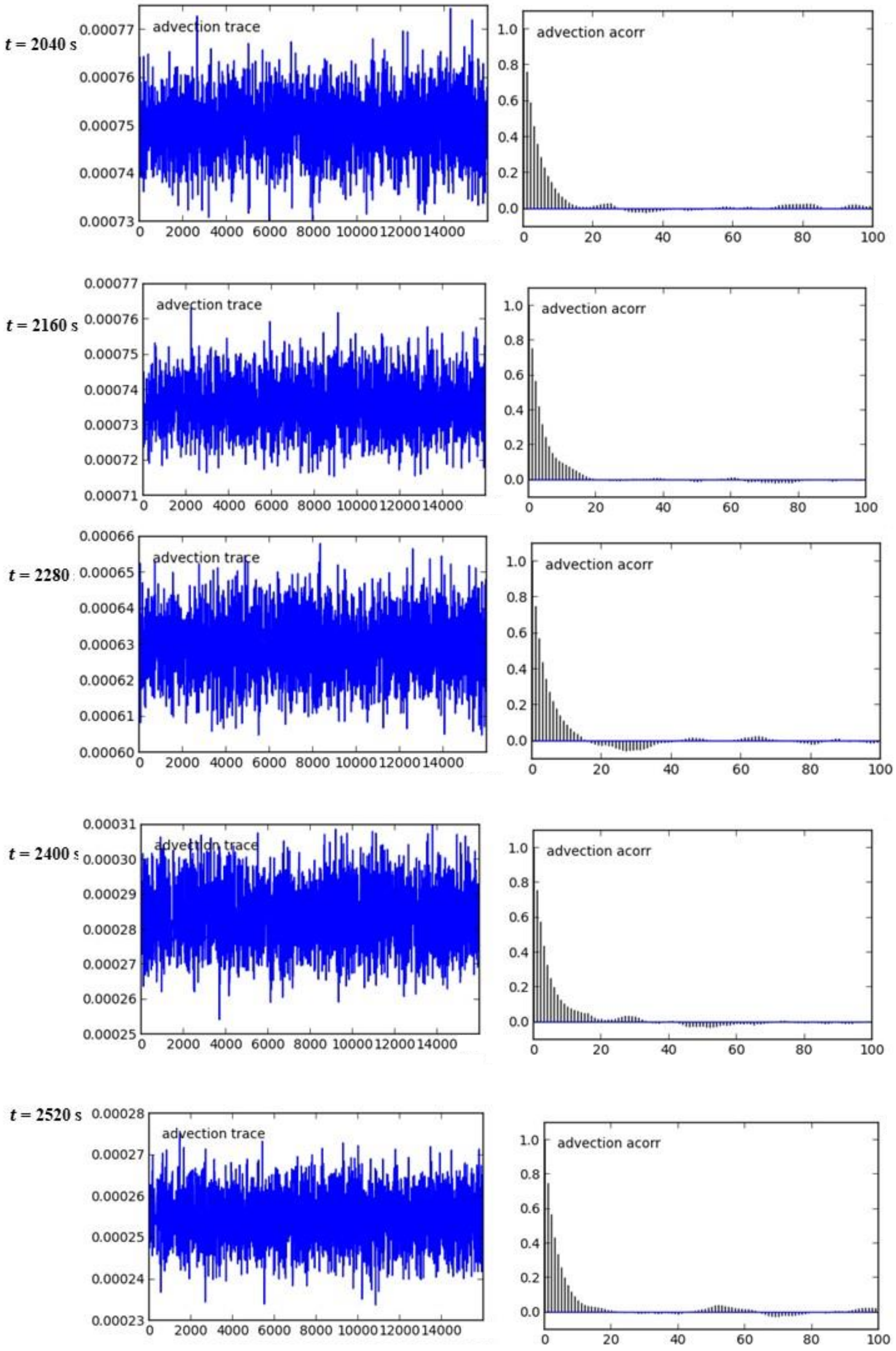

Dataset I

Experimental noise $(a.u.)$

$t = 2040$ s

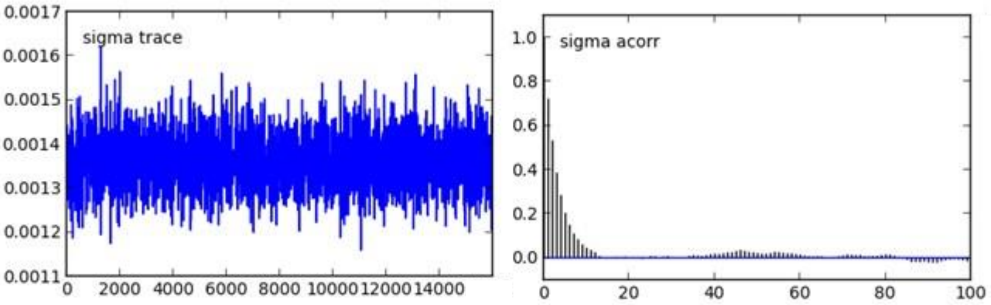

$t = 2160$ s

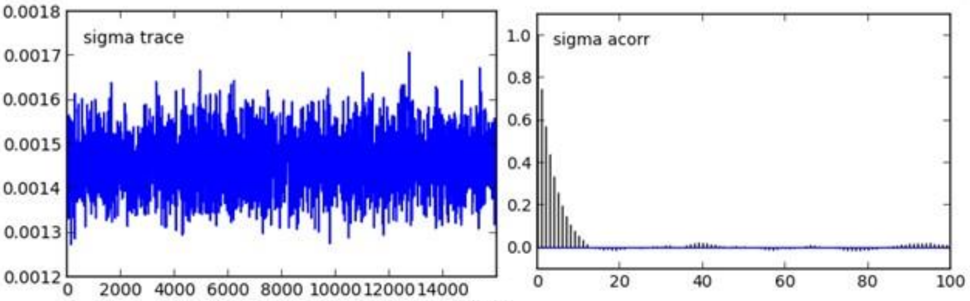

$t = 2280$ s

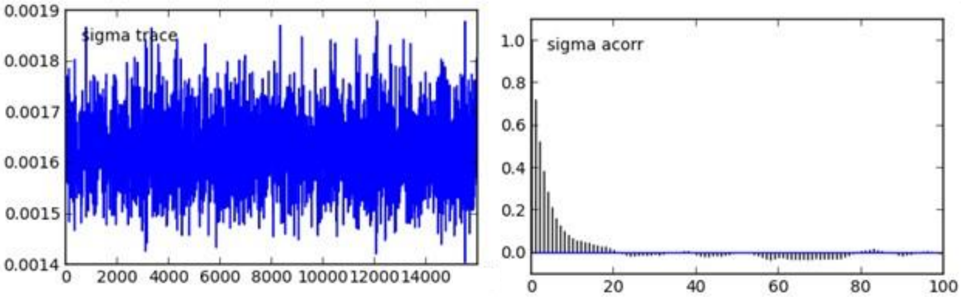

$t = 2400$ s

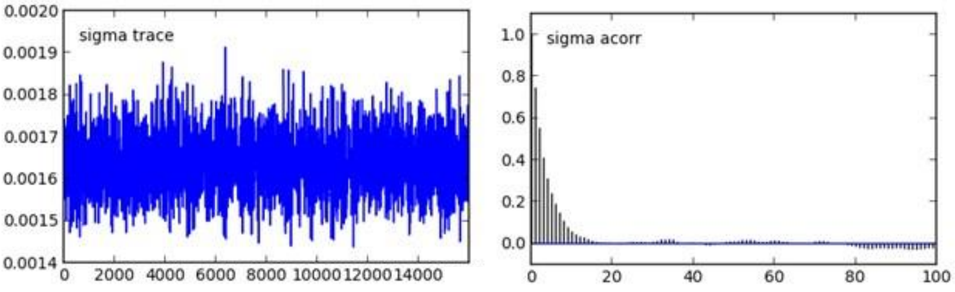

$t = 2520$ s

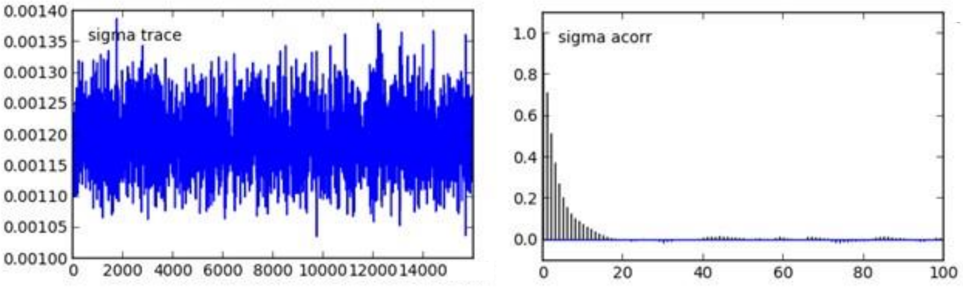

Dataset II

Diffusivity $D(mm^2/s)$

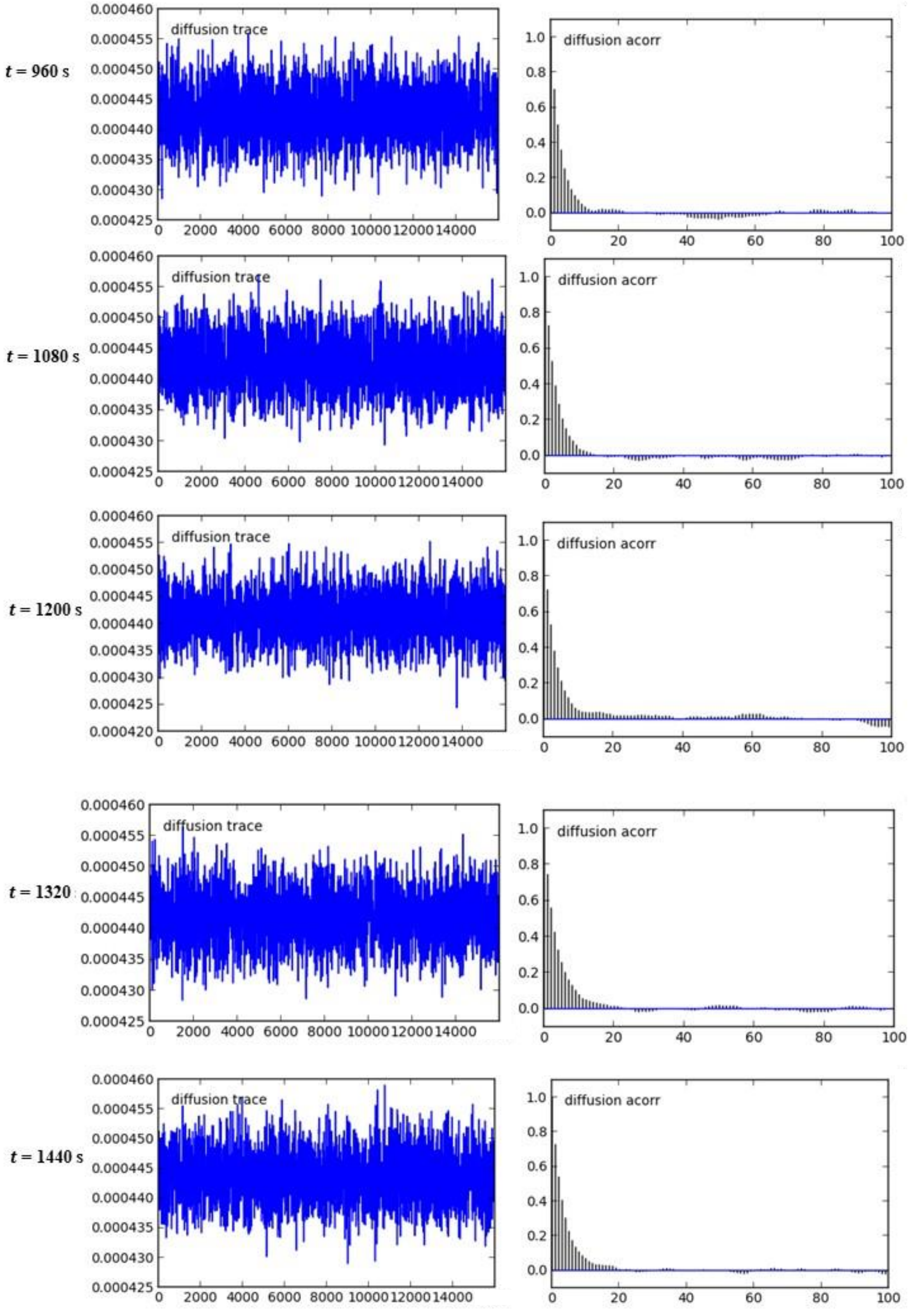

Dataset II

Advection $\boldsymbol{u}$ (mm/s)

$t = 960$ s

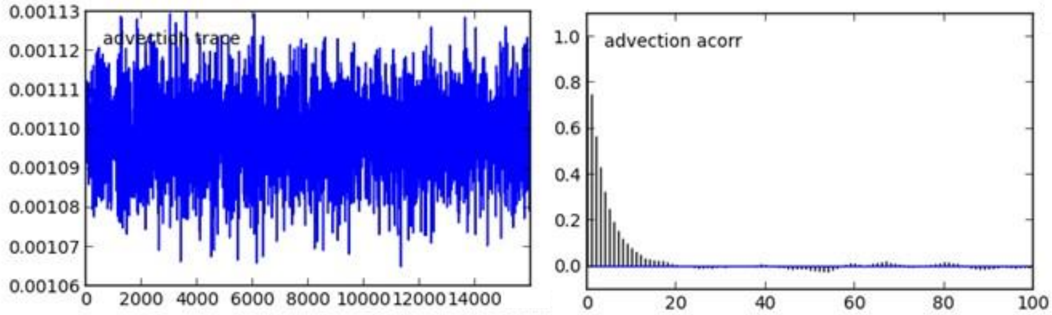

$t = 1080$ s

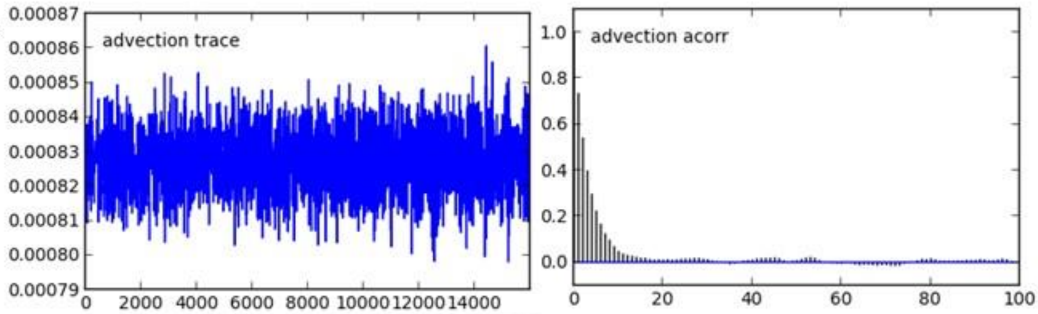

$t = 1200$ s

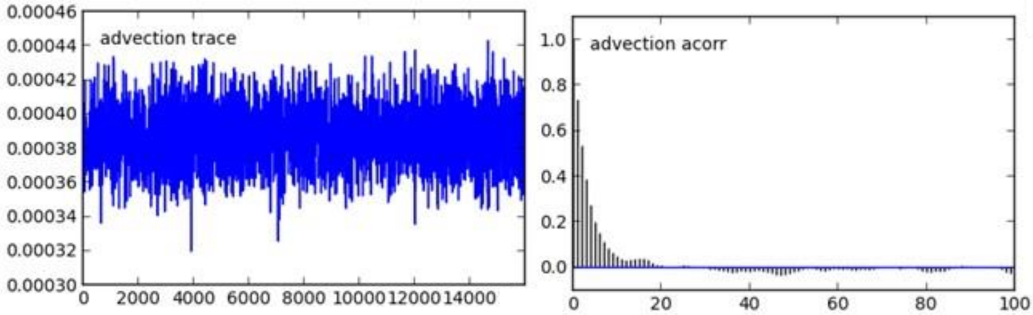

$t = 1320$ s

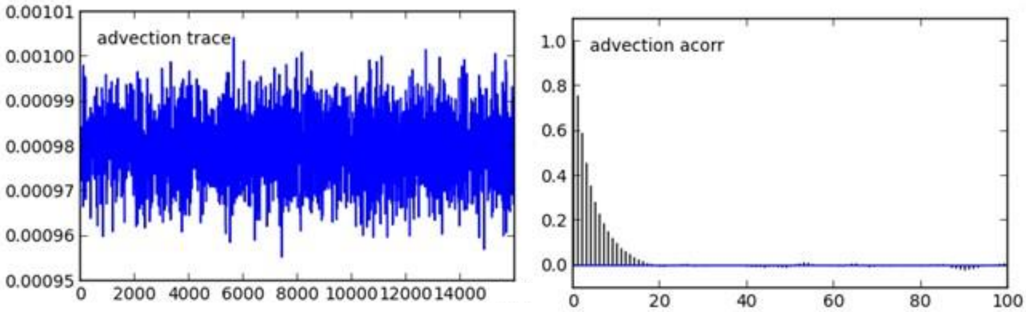

$t = 1440$ s

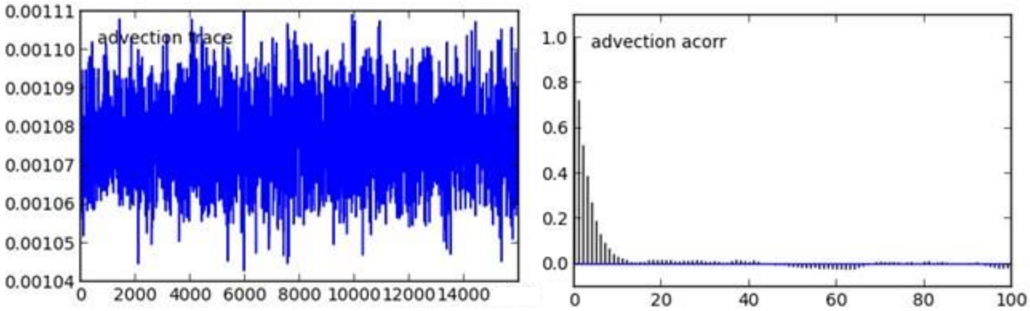

Dataset II

Experimental noise $(a.u.)$

$t = 960$ s

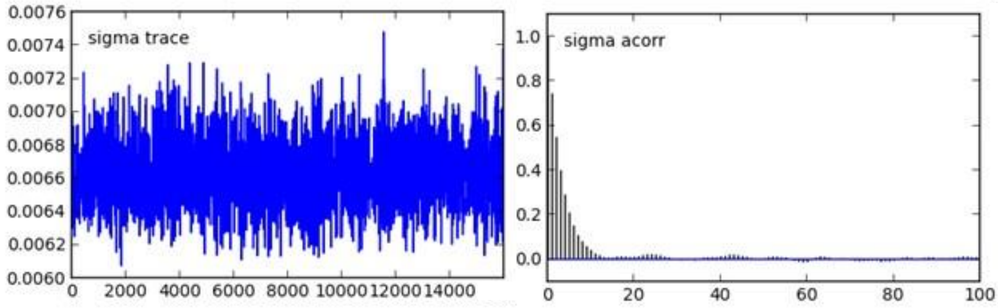

$t = 1080$ s

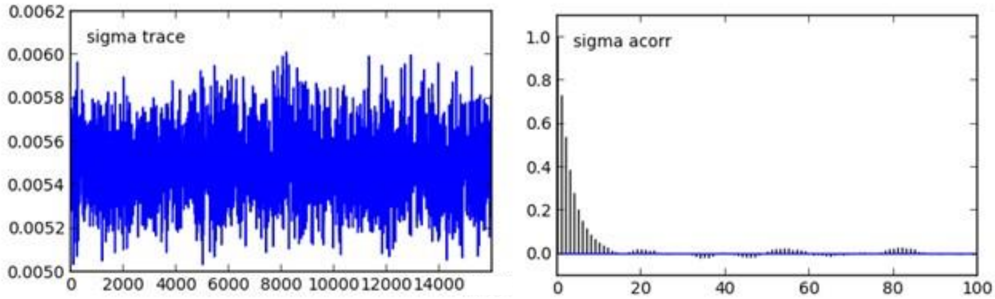

$t = 1200$ s

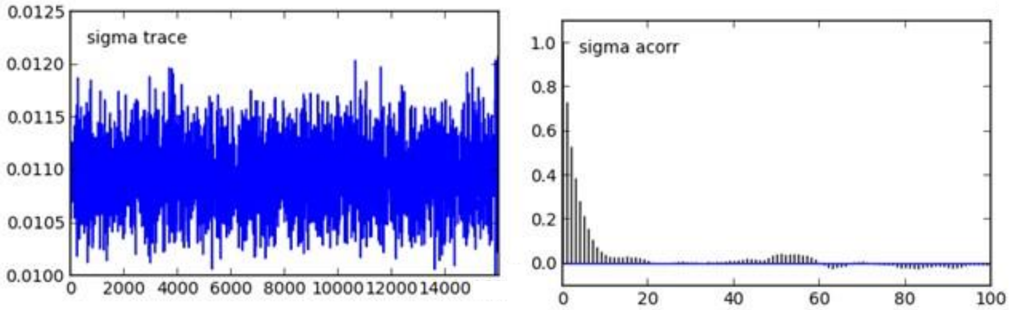

$t = 1320$ s

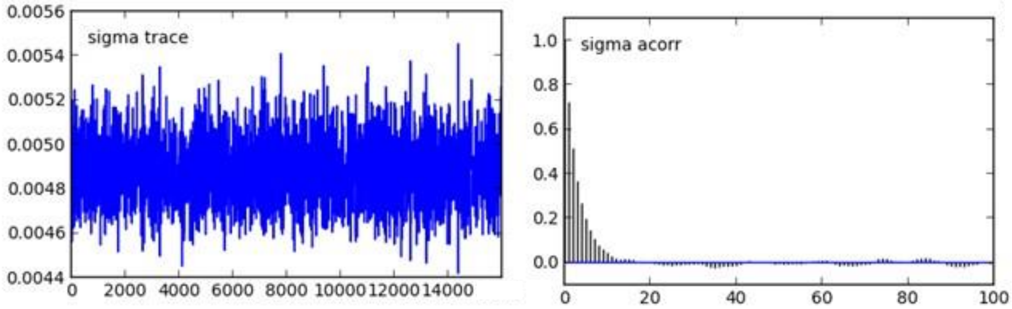

$t = 1440$ s

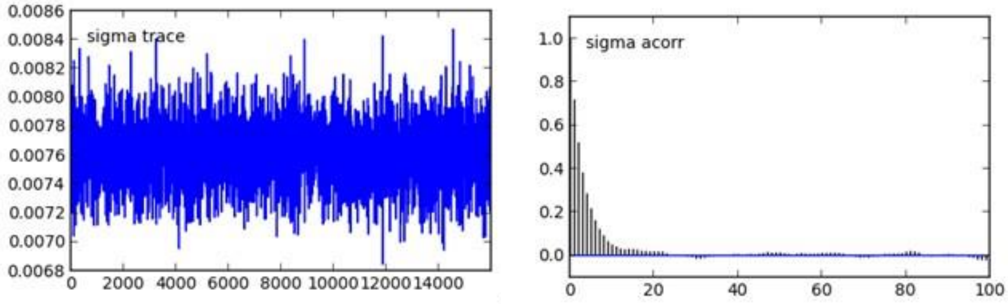

Supplement: Supplementary file 1 [file Data_Sheet_1.pdf]
